# Diagnostic value of smartphone in obstructive sleep apnea syndrome: A systematic review and meta-analysis

**DOI:** 10.1371/journal.pone.0268585

**Published:** 2022-05-19

**Authors:** Do Hyun Kim, Sung Won Kim, Se Hwan Hwang

**Affiliations:** 1 Department of Otolaryngology-Head and Neck Surgery, Seoul St. Mary’s Hospital, College of Medicine, The Catholic University of Korea, Seoul, Korea; 2 Department of Otolaryngology-Head and Neck Surgery, Bucheon St. Mary’s Hospital, College of Medicine, The Catholic University of Korea, Seoul, Korea; Charité - Universitätsmedizin Berlin, GERMANY

## Abstract

**Objectives:**

To assess the diagnostic utility of smartphone-based measurement in detecting moderate to severe obstructive sleep apnea syndrome (OSAS).

**Methods:**

Six databases were thoroughly reviewed. Random-effect models were used to estimate the summary sensitivity, specificity, negative predictive value, positive predictive value, diagnostic odds ratio, summary receiver operating characteristic curve and measured the areas under the curve. To assess the accuracy and precision, pooled mean difference and standard deviation of apnea hypopnea index (AHI) between smartphone and polysomnography (95% limits of agreement) across studies were calculated using the random-effects model. Study methodological quality was evaluated using the QUADAS-2 tool.

**Results:**

Eleven studies were analyzed. The smartphone diagnostic odds ratio for moderate-to-severe OSAS (apnea/hypopnea index > 15) was 57.3873 (95% confidence interval [CI]: [34.7462; 94.7815]). The area under the summary receiver operating characteristic curve was 0.917. The sensitivity, specificity, negative predictive value, and positive predictive value were 0.9064 [0.8789; 0.9282], 0.8801 [0.8227; 0.9207], 0.9049 [0.8556; 0.9386], and 0.8844 [0.8234; 0.9263], respectively. We performed subgroup analysis based on the various OSAS detection methods (motion, sound, oximetry, and combinations thereof). Although the diagnostic odds ratios, specificities, and negative predictive values varied significantly (all p < 0.05), all methods afforded good sensitivity (> 80%). The sensitivities and positive predictive values were similar for the various methods (both p > 0.05). The mean difference with standard deviation in the AHI between smartphone and polysomnography was -0.6845 ± 1.611 events/h [-3.8426; 2.4735].

**Conclusions:**

Smartphone could be used to screen the moderate-to-severe OSAS. The mean difference between smartphones and polysomnography AHI measurements was small, though limits of agreement was wide. Therefore, clinicians should be cautious when making clinical decisions based on these devices.

## Introduction

Obstructive sleep apnea syndrome (OSAS) is a disorder associated with periodic breathing cessation, significantly reducing the quality of life, and increasing cardiovascular disease and mortality [1, 2]. The prevalence of OSAS is 9 to 38% in the general population and has increased in recent years [2, 3]. Attended polysomnography (PSG) in a sleep laboratory is currently the gold-standard tool for OSAS diagnosis. PSG data are used to assess apnea/hypopnea events, oxygen desaturations, and arousal frequency. The number of apnea and hypopnea events per hour (the apnea/hypopnea index [AHI]) is a measure of sleep apnea severity [4]. However, the costs of a special room, monitoring facilities, and specialized personnel limit access for many potential patients. Furthermore, PSG evaluations are usually limited to one night, associated with false- negatives; significant variations in OSAS severity have been observed over multiple nights. OSAS must become easily and cheaply detectable [5]. Today, smartphones can collect sound, movement, and oximeter data [6–8]. Therefore, there were reports comparing PSG and portable devices to evaluate the sleep environment in a more patient-friendly environment, away from the inconvenient and artificial sleep environment of the PSG setting [5–15]. Among portable devices, many recent apps for sleep tests use a smartphone computer and sensors [16–19]. Currently, no meta-analysis either supports or does not support the suggestion that smartphones could effectively screen for OSA. Therefore, we performed a meta-analysis that can intuitively compare and evaluate the diagnostic accuracy and utility of smartphones using various sensing devices in terms of OSAS screening.

## Materials and methods

### Search strategy and study selection

We retrieved clinical data from PubMed, Embase, the Web of Science, SCOPUS, the Cochrane Central Register of Controlled Trials, and Google Scholar from the dates of inception to May 2021. The population, intervention, comparison, and outcome (PICO) parameters were: P, patients with suspected OSA who evaluated sleep disorder with PSG and smartphone; I, biological data measured by the smartphone; C, sleep data measured by PSG; and O, the AHI. Only papers written in English were considered. The search terms were: “sleep disturbed breathing,” “obstructive sleep apnea,” “smartphone,” “polysomnography,” “mobile,” and “screening.” The reference lists were checked to ensure that no relevant studies were omitted. The titles and abstracts of all candidate studies were systematically reviewed by two independent reviewers.

### Selection criteria

**Fig 1:**
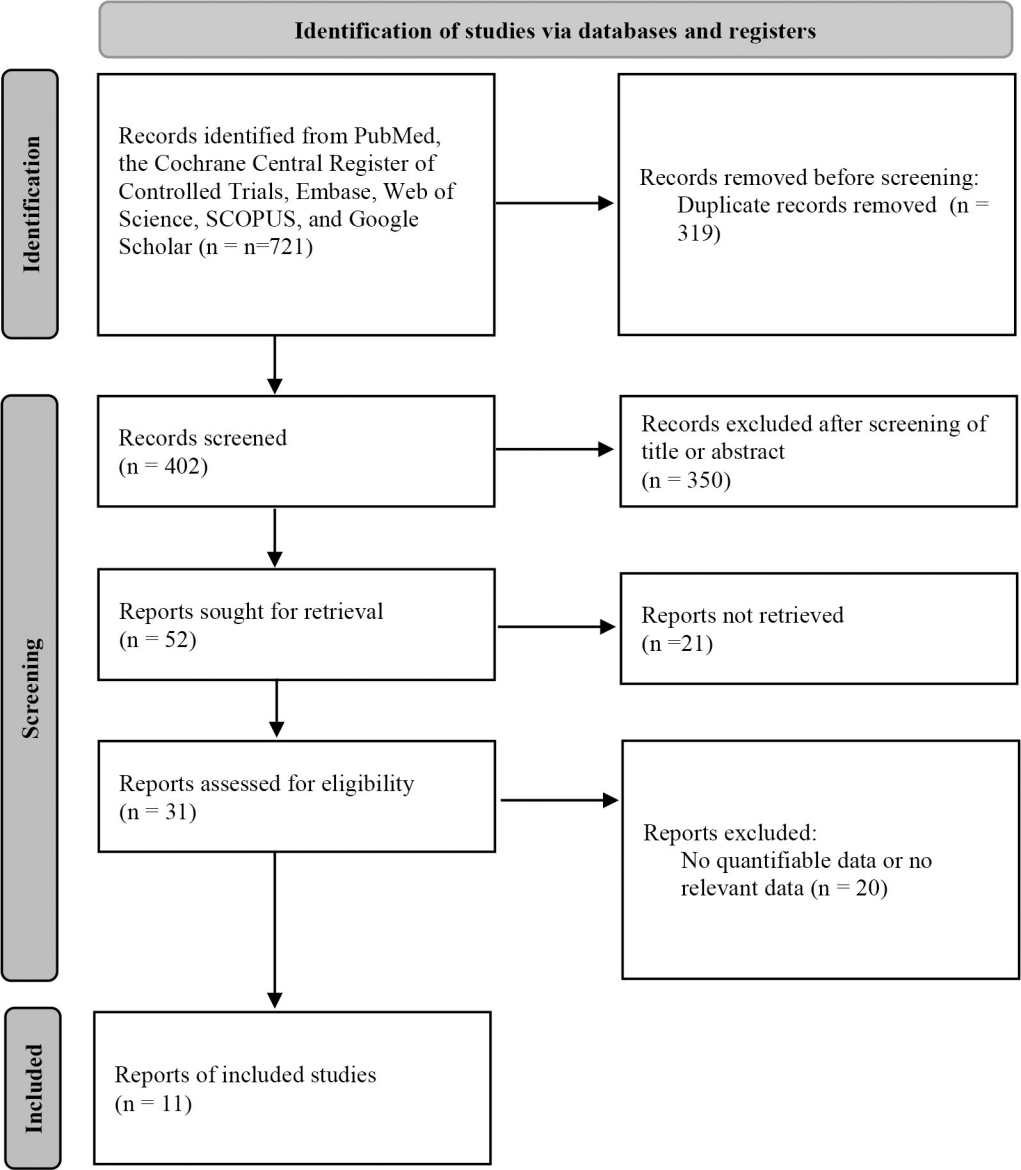
Flowchart of article selection (PRISMA).

The inclusion criteria were: 1) a study analyzing sleep with mobile phone device and/or accessories; 2) a cohort study; 3) a comparison between a smartphone and PSG (the reference); and 4) an article containing sensitivity and specificity values data. The exclusion criteria were: 1) case reports; 2) review articles; 3) diagnosis or screening of OSAS with other portable devices except smartphone; and 4) insufficient data. The search strategy is shown in Fig 1.

### Data extraction and bias assessment

Two reviewers (SWK and SHH) independently selected and compared eligible studies, then extracted data using standardized forms. We collected study identification, publication year, study design, enrolled number of patients, mean AHI, Sex ratio, apnea detecting mechanism, cut off values of AHI, and 2x2 table outcomes. At each stage, the papers selected by two reviewers were compared, and if the selected articles were inconsistent, a final decision was made through panel discussion with the third reviewer (DHK). The bias assessment was performed in the same way. The search terms and queries were presented in S1 Table.

We analyzed the predictive power of sleep analysis by a smartphone (i.e., the diagnostic odds ratio [DOR]), then constructed summary receiver operating characteristic (SROC) curves and measured the areas under the curves (AUCs) [5–15]. Moderate to severe OSAS (AHI ≥ 15) was defined based on PSG. True-positive, true-negative, false-positive, and false-negative values were collected for the calculation of AUCs and DORs. To calculate the accuracy and precision of smartphone compared to PSG, we extracted mean difference and standard deviation (SD) of mean differences between the PSG-AHI and smartphone-AHI measurements from a single study. They were pooled in meta-analysis to yield a summary estimate (weighted mean difference) [5, 6, 13]. The quality of each study was analyzed using the Quality Assessment of Diagnostic Accuracy Studies ver. 2 (QUADAS-2) tool [20].

### Statistical analysis and measurement of outcomes

Meta-analysis was conducted using R statistical software (R Foundation for Statistical Computing, Vienna, Austria [version 3.6.3]). Explore the cause of significant between-study heterogeneity among the studies, subgroup analyses were performed (motion, sound, oximetry, and combinations thereof). We generated forest plots of sensitivities, specificities, and negative predictive values, as well as SROC curves.

Heterogeneity was calculated with the I^2^ test: The I^2^ test describes the rate of variation across studies caused by heterogeneity rather than probabilistic chance; the measure ranges from 0 (no heterogeneity) to 100 (maximum heterogeneity). When significant heterogeneity among outcomes was found (defined as I^2^ > 50), the random-effects model was used according to DerSimonian-Laird. Those outcomes that did not present a significant level of heterogeneity (I^2^ < 50) were analyzed with the fixed-effects model. The fixed-effects model uses the inverse variance approach, and it is assumed that all studies come from a common population. Sensitivity analyses were performed to determine the effects of individual studies on the overall meta-analysis results.

We used Begg’s funnel plot and Egger’s test simultaneously to detect publication bias. The trim-and-fill method also was done to indicate the significance of publication bias as well as provide bias-adjusted results.

## Results

In total, 11 studies with 1,644 participants were included. Study characteristics and bias assessments are presented in S2 and S3 Tables. Egger’s test (*p*> 0.05) and Begg’s funnel plot (S1 Fig) on these measurements suggested that a bias source was not evident in this sample of studies.

### Diagnostic accuracy of smartphones in terms of moderate-to-severe obstructive sleep apnea syndrome

**Fig 2:**
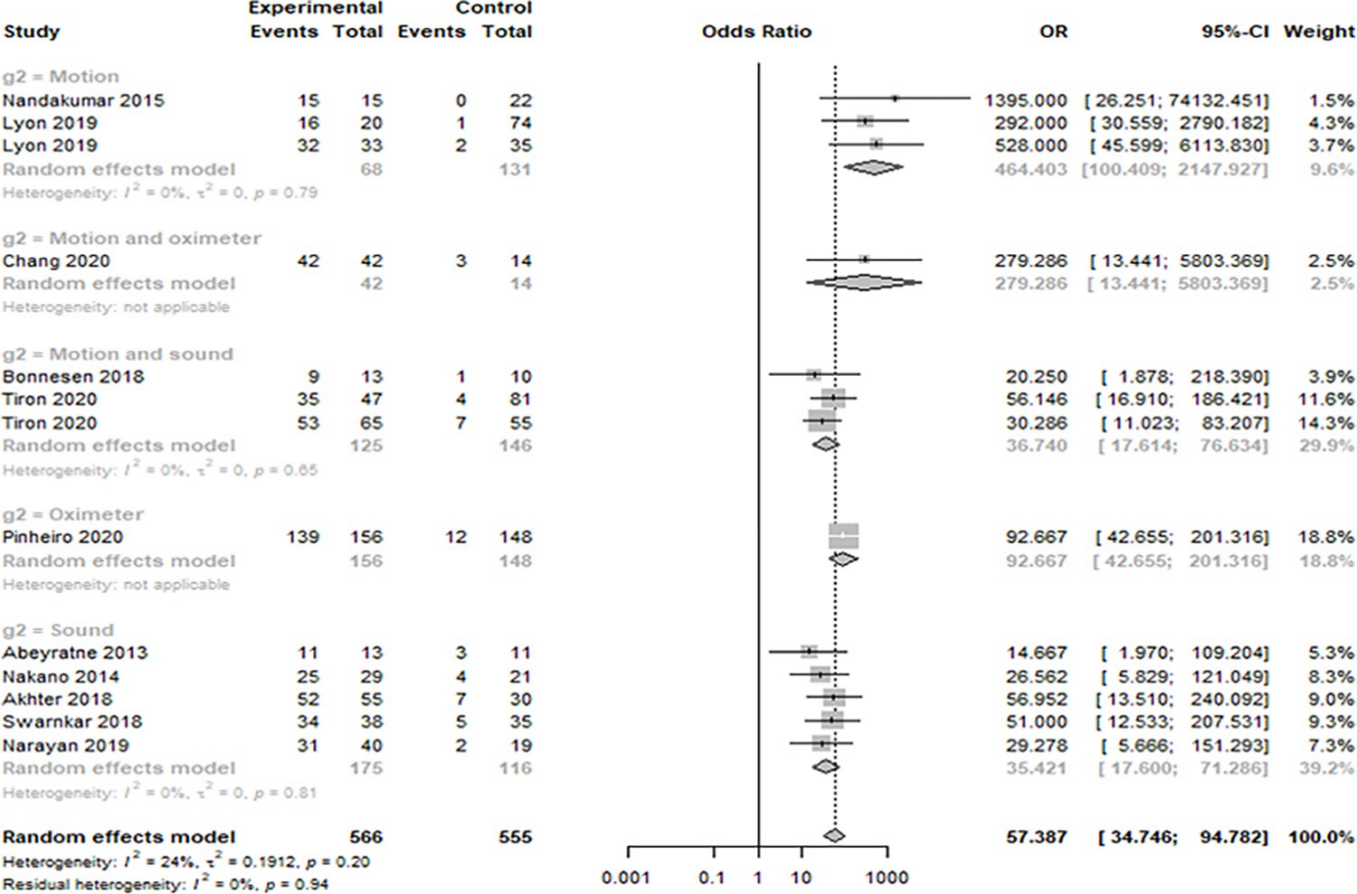
Forest plot of the diagnostic odds ratios of the included studies.

**Fig 3:**
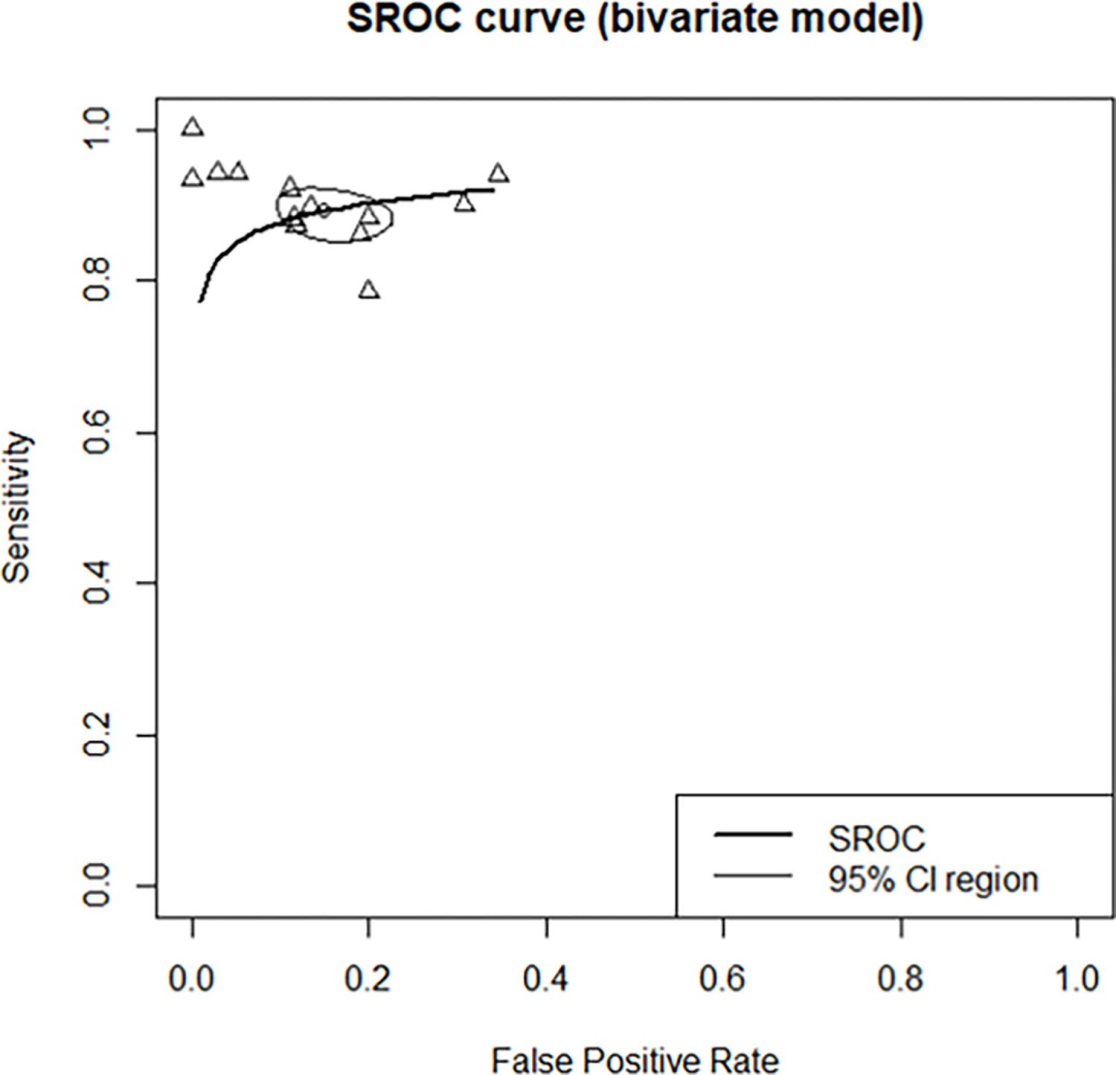
Area under the summary receiver operating characteristic curves of included studies.

**Fig 4:**
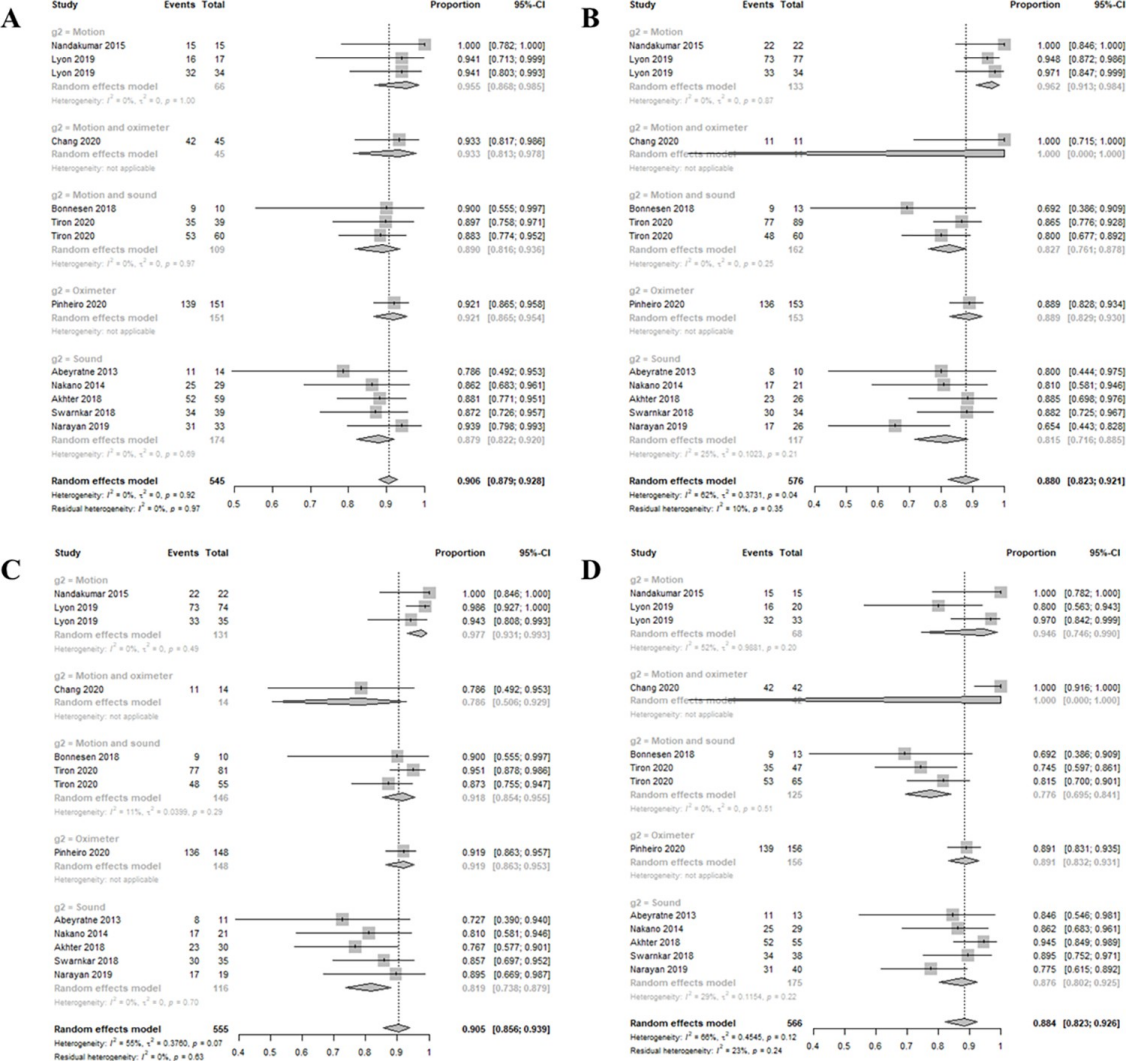
Forest plots of the sensitivities (A), specificities (B), negative predictive values (C), and positive predictive values (D) of the included studies.

**Fig 5:**
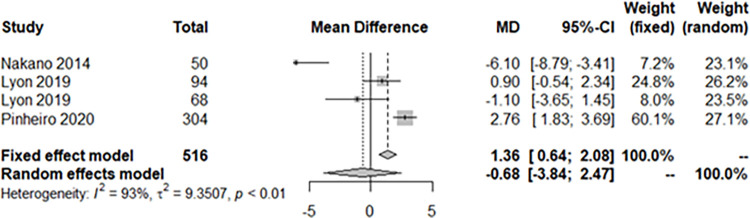
Overall pooled random-effects mean difference of apnea hypopnea index between smartphone and polysomnography across studies.

Eleven studies were analyzed. The smartphone DOR for moderate-to-severe OSAS (AHI > 15) was 57.3873 (95% confidence interval [CI]: 34.7462; 94.7815, I^2^ = 24.3%) (Fig 2). The area under the SROC curve was 0.917 (Fig 3). The correlation between sensitivity and the false-positive rate was 0.137, indicating that heterogeneity was absent. The sensitivity, specificity, negative predictive value, and positive predictive value were 0.9064 ([0.8789; 0.9282], I^2^ = 0.0%), 0.8801 ([0.8227; 0.9207], I^2^ = 61.7%), 0.9049 ([0.8556; 0.9386], I^2^ = 54.9%), and 0.8844 ([0.8234; 0.9263], I^2^ = 66.3%), respectively (Fig 4). The overall pooled random-effects mean difference (smartphone—polysomnography) and SD were -0.6845 ([-3.8426; 2.4735], I^2^ = 92.9%) (Fig 5).

Subgroup analysis was performed according to the mode of detection of apnea severity (using motion, sound, oximetry, and combinations thereof) because high heterogeneity in terms of diagnostic accuracy was evident (Table 1). In terms of specificity, motion or oximetry methods (motion 96%; motion and oximetry 100%; oximetry 88%) were better than the other methods (82 and 81%; p = 0.0085). In terms of the negative predictive value, motion or oximetry methods (motion 97%; motion and sound 92%; and oximeter 92%) were better than the other methods (78 and 81%; p = 0.0015). In terms of the DOR, motion or oximetry methods (motion 464.4033; motion and oximetry 279.2857; and oximetry 92.6667) were better than the other methods (36.7401 and 35.4208; p = 0.0117). In contrast, the specificity and both negative and positive predictive values were similar (80–100%, all p > 0.05) for all methods. However, although only single study used only oximetry, it was clear that smartphones afforded good diagnostic accuracies (in terms of sensitivity, specificity, negative predictive value, and positive predictive value; all 80–100%) when screening for moderate-to-severe OSAS.

**Table 1:** Subgroup analysis according to detection method.

Subgroup	Study (n)	DOR [95% CIs]; I^2^	Sensitivity [95% CIs]; I^2^	Specificity [95% CIs]; I^2^	NPV; I^2^	PPV; I^2^
**methods of screening the moderate to severe OSAS**	11	57.3873 [34.7462; 94.7815]; 24.3%	0.9064 [0.8789; 0.9282]; 0.0%;	0.8801 [0.8227; 0.9207]; 61.7%;	0.9049 [0.8556; 0.9386]; 54.9%	0.8844 [0.8234; 0.9263]; 66.3%
Motion	3	464.4033 [100.4086; 2147.9273]; 0.0%	0.9545 [0.8683; 0.9853]; 0.0%	0.9624 [0.9129; 0.9843]; 0.0%	0.9771 [0.9314; 0.9926]; 0.0%	0.9460 [0.7465; 0.9905]; 52.1%
Motion and oximeter	1	279.2857 [13.4406; 5803.3689]; NA	0.9333 [0.8127; 0.9783]; NA	1.0000 [0.0000; 1.0000]; NA	0.7857 [0.5057; 0.9293]; NA	1.0000 [0.0000; 1.0000]; NA
Motion and sound	3	36.7401 [17.6141; 76.6336]; 0.0%	0.8899 [0.8161; 0.9364]; 0.0%	0.8272 [0.7610; 0.8779]; 0.0%	0.9177 [0.8539; 0.9551]; 10.6%	0.7760 [0.6947; 0.8406]; 0.0%
Oximeter	1	92.6667 [42.6548; 201.3163]; NA	0.9205 [0.8653; 0.9543]; NA	0.8889 [0.8285; 0.9298]; NA	0.9189 [0.8627; 0.9534];NA	0.8910 [0.8317; 0.9312]; NA
Sound	5	35.4208 [17.5999; 71.2864]; 0.0%	0.8793 [0.8220; 0.9200]; 0.0%	0.8149 [0.7157; 0.8851]; 24.8%	0.8190 [0.7382; 0.8789]; 0.0%	0.8759 [0.8023; 0.9247]; 28.7%

DOR; diagnostic odds ratio, CI; confidence interval, NPV; negative predictive value, PPV; positive predictive value, AUC; area under the curve.

### Sensitivity analyses

We evaluated differences in pooled estimates by repeating the meta-analysis, omitting study one at a time. All results were consistent with the above results (S2 Fig).

## Discussion

In this study, the smartphone diagnostic accuracy exhibited a pooled sensitivity of 0.90, a pooled specificity of 0.88, a pooled negative predictive value of 0.90, a pooled positive predictive value of 0.88, and an AUC of 0.92. All AUCs under SROC curves were 0.9–1.00, suggesting excellent diagnostic accuracy. The sensitivity in terms of moderate-to-severe OSA was good (90%). The specificity in terms of the absence of moderate-to-severe OSA was 88%; the false-negative rate was thus very low. These results mean that smartphone-based OSAS screening would be useful for patients with moderate-to-severe OSAS. The high negative predictive value suggests that only 10% of smartphone-positive patients would have false-positive diagnoses, compared with patients diagnosed on the basis of clinical examination or history-taking.

Attended full PSG is the gold-standard tool for OSAS diagnosis. However, the patient must sleep in an unfamiliar specialized room with 22 wires attached; these collect neurological, cardiac, and respiratory data. The process can cause serious discomfort and anxiety; the patient may not be able to sleep as usual. Therefore, some clinics prescribe sleeping pills, which distort analysis. Moreover, few hospitals feature full PSG, particularly in rural areas, and PSG is expensive [21, 22]. There is an urgent need for portable devices that are accurate, convenient, and measure only key biological signals [23, 24].

Smartphones feature various apps [25]. Internal sensors and external (connectable) devices measure blood oxygen, pulse, body movement (using accelerometers or sonars), and breath sounds during sleep [12]. Studies using oximeter/accelerometer combinations to diagnose sleep apnea found that body position data aided in respiratory movement assessment [26, 27]. Breathing sounds during sleep also aid in OSAS diagnosis. Snoring differs between healthy people and sleep apnea patients; the noises alone accurately separate the groups [28]. In addition, various recent apps feature algorithms analyzing oxygen saturation, body position during sleep, and sleep breathing sounds [5–10, 12, 14, 15, 29]. Furthermore, because smartphones use motion, sound, oximetry, and combinations thereof to detect abnormal sleep, we evaluated the effects of the various methods on diagnostic accuracy; we performed subgroup analysis.

Recent studies have compared the reliabilities of such apps to the reliability of PSG [5, 8, 16–19]. Because moderate-to-severe OSA ([AHI ≥ 15/h) is associated with high risks of cardiovascular morbidity and mortality, and thus requires treatment [30], many studies have sought to clinically validate apps by screening for such OSA [5–10, 12, 14, 15, 29].

Smartphones may usefully screen for OSAS among individuals who may be unaware of a problem, such as singles with no consistent bed partners. It is thus easy to screen patients with high risks of cardiovascular and cerebrovascular diseases because of hypoxia during sleep [8]. In addition, smartphones could be useful to follow-up patients wearing oral devices or who have undergone upper airway surgery. According to the recent development of devices and algorithms, the Respiratory Event Index (REI), which performs automatic scoring by coupling heart rate variability and oxygen saturation changes using a device such as a smartphone, has a good correlation with AHI and can be used as a useful tool to evaluate the patient’s hypoxic burden [31–34]. However, the apps allow only self-problem checking, not counseling. Additionally, clinicians should be aware that smartphones simply reveal good correlations between sleep data and the AHI, but do not integrate all of the important PSG findings. For example, OSAS is a heterogeneous disease that can show multiple phenotypes [35, 36]. When measured through smartphones, patients with disrupted sleep with insomnia might not be measured properly.

On subgroup analysis of the detection methods, all approaches exhibited similar (and good) sensitivities and specificities (80–100%), but methods employing pulse oximetry or motion detection tended to be more diagnostically accurate. A pulse oximeter measures blood oxygen levels non-invasively and continuously [37]. A strong correlation between the AHI and oxygen desaturation was reported in a group of patients with suspected sleep apnea [38]. Moreover, most movement during sleep reflects the respiratory efforts of the torso, which are critical for sleep apnea detection; movement-based apnea estimation is strongly correlated with PSG data, particularly for patients with moderate-to-severe OSA [39]. The use of pulse oximetry or motion detection is optimal.

Another measurement of this meta-analysis assessed the agreement between smartphone and PSG AHI measurements. The mean difference was small (around -0.7) but the limits of agreement was wide (around 6.2). It could mean that smartphone measured AHI seemed significantly close to the true value from PSG but the variability of repeated values due to random error in the smartphone looked considerable. An AHI measured by smartphone may be accurate but imprecise meaning that resultant values are close to the true value but can be inconsistent. Therefore, clinicians need to be cautious when making clinical decision. Also, because the results were based on only four studies, more work is required to support them.

This study had some limitations. First, we analyzed only a limited number of studies, despite extensive searching. To our knowledge, there have been few relevant studies. Therefore, more work is required. Second, high heterogeneity was evident in studies evaluating whether smartphones could screen for moderate-to-severe OSAS, reflecting the use of different detection methods in and OSAS definitions. Also, PSG used as a control group was mostly type I full PSG, but the type was not specified in three studies. It is possible that an unidentified PSG may have influenced the results. These must be standardized in future studies.

Based on our results, and despite the limitations, smartphones serve as useful adjuncts when screening for moderate-to-severe OSAS. Smartphones cannot replace PSG. However, smartphones greatly aid in large-scale screening, thus detecting OSAS patients who are prone to misdiagnosis and OSAS patients in regions with poor or inaccessible medical facilities; they can avoid the need for expensive equipment and rooms, as well as specialized personnel.

## Conclusion

Smartphone-based OSAS screening would be useful for patients with moderate-to-severe OSAS. While various OSAS detection methods exhibited similar sensitivity and specificity, using pulse oximetry or motion detection tended to be more accurate diagnostically. Smartphone-based OSAS screening cannot replace PSG, but greatly aids in large-scale screening, thus detecting OSAS patients who are prone to misdiagnosis and OSAS patients in regions with poor or inaccessible medical facilities. However, assessing the agreement between smartphone and PSG AHI measurements, the wide limits of agreement mean clinicians should be cautious when making clinical decisions based on these devices. Further studies are needed, and smartphone detection methods must be strictly standardized.

## Supporting information

S1 Checklist PRISMA-DTA checklist. (DOC)

S1 Table Search terms and queries. (DOCX)

S2 Table Study characteristics. (DOCX)

S3 Table Methodological qualities of all included studies. (DOCX)

S1 Fig Begg’s funnel plot analyses for sensitivity (A), specificity (B), negative predictive value (C), positive predictive value (D), and diagnostic odds ratio (E).
(TIF)

S2 Fig Sensitivity analyses for sensitivity (A), specificity (B), negative predictive value (C), positive predictive value (D), and diagnostic odds ratio (E).
(TIF)
