# Supplementary material for: Diagnostic value of smartphone in obstructive sleep apnea syndrome: A systematic review and meta-analysis
Source: PLoS One. 2022 May 19;17(5):e0268585. doi: 10.1371/journal.pone.0268585 (PMC9119483; doi:10.1371/journal.pone.0268585)
Supplement: S1 Table — (DOCX) [file pone.0268585.s002.docx]

**S1 Table. Search terms and queries.**

| Database | Search | Search terms/queries |
| --- | --- | --- |
| PubMed | #1 | "cell phone"[MeSH Terms] OR "smartphone"[MeSH Terms] OR "polysomnography"[MeSH Terms] |
|  | #2 | "cell phone"[Text Word] OR "mobilephone"[Text Word] OR "smartphone"[Text Word] OR "polysomnograph*"[Text Word] OR "somnograph*"[Text Word] OR "sleep monitoring"[Text Word] OR "apnea monitoring"[Text Word] |
|  | #3 | #1 OR #2 |
|  | #4 | "sleep apnea, obstructive"[MeSH Terms] OR "sleep apnea syndromes"[MeSH Terms] |
|  | #5 | "obstructive sleep apnea*"[Text Word] OR "sleep apnea syndrome*"[Text Word] OR "sleep hypopnea*"[Text Word] OR "sleep apnea*"[Text Word] OR "hypersomnia with periodic respiration"[Text Word] OR "sleep disturbed breathing"[Text Word] OR "sleep disordered breathing"[Text Word] |
|  | #6 | #4 OR #5 |
|  | #7 | ("screening*"[Text Word] OR "diagnos*"[Text Word]) AND ("accurac*"[Text Word] OR "sensitivit*"[Text Word]) |
|  | #8 | #3 AND #6 AND #7 |
|  | #9 | #3 AND #6 AND #7 Filters: English |
|  |  |  |
| Database | Search | Search terms/queries |
| EMBASE | #1 | mobile phone'/exp OR 'polysomnography'/exp |
|  | #2 | cell phone':ab,ti OR 'mobilephone':ab,ti OR 'smartphone':ab,ti OR 'polysomnograph*':ab,ti OR 'somnograph*':ab,ti OR 'sleep monitoring':ab,ti OR 'apnea monitoring':ab,ti |
|  | #3 | #1 OR #2 |
|  | #4 | sleep disordered breathing'/exp |
|  | #5 | obstructive sleep apnea*':ab,ti OR 'sleep apnea syndrome*':ab,ti OR 'sleep hypopnea*':ab,ti OR 'sleep apnea*':ab,ti OR 'hypersomnia with periodic respiration':ab,ti OR 'sleep disturbed breathing':ab,ti OR 'sleep disordered breathing':ab,ti |
|  | #6 | #4 OR #5 |
|  | #7 | diagnostic accuracy'/exp |
|  | #8 | ('screening*':ab,ti OR 'diagnos*':ab,ti) AND ('accurac*':ab,ti OR 'sensitivit*':ab,ti) |
|  | #9 | #7 OR #8 |
|  | #10 | #3 AND #6 AND #9 |
|  | #11 | #3 AND #6 AND #9 AND [english]/lim |
|  |  |  |
| Database | Search | Search terms/queries |
| Cochrane Library | #1 | MeSH descriptor: [Cell Phone] explode all trees |
|  | #2 | MeSH descriptor: [Smartphone] explode all trees |
|  | #3 | MeSH descriptor: [Polysomnography] explode all trees |
|  | #4 | ('cell phone' OR 'mobilephone' OR 'smartphone' OR 'polysomnograph*' OR 'somnograph*' OR 'sleep monitoring' OR 'apnea monitoring'):ti,ab,kw |
|  | #5 | #1 OR #2 OR #3 OR #4 |
|  | #6 | MeSH descriptor: [Sleep Apnea, Obstructive] explode all trees |
|  | #7 | MeSH descriptor: [Sleep Apnea Syndromes] explode all trees |
|  | #8 | ('obstructive sleep apnea*' OR 'sleep apnea syndrome*' OR 'sleep hypopnea*' OR 'sleep apnea*' OR 'hypersomnia with periodic respiration' OR 'sleep disturbed breathing' OR 'sleep disordered breathing'):ti,ab,kw |
|  | #9 | #6 OR #7 OR #8 |
|  | #10 | ('screening*' OR 'diagnos*'):ti,ab,kw AND ('accurac*' OR 'sensitivit*'):ti,ab,kw |
|  | #11 | #5 AND #9 AND #10 |
|  |  |  |
| Database | Search | Search terms/queries |
| Web of Science | #1 | ALL=('cell phone' OR 'smartphone' OR 'mobilephone' OR 'polysomnograp*' OR 'somnograph*' OR 'sleep monitoring' OR 'apnea monitoring' ) |
|  | #2 | ALL=('obstructive sleep apnea*' OR 'sleep apnea syndrome*' OR 'sleep hypopnea*' OR 'sleep apnea*' OR 'hypersomnia with periodic respiration' OR 'sleep disturbed breathing' OR 'sleep disordered breathing') |
|  | #3 | ALL=((screening*' OR 'diagnos*') AND ('accurac*' OR 'sensitivit*')) |
|  | #4 | #1 AND #2 AND #3 |
|  | #5 | #1 AND #2 AND #3 /English |
|  |  |  |
| Database | Search | Search terms/queries |
| Google Scholar | #1 | ('cell phone' OR 'smartphone' OR 'mobilephone' OR 'polysomnograp*' OR 'somnograph*' OR 'sleep monitoring' OR 'apnea monitoring' ) AND ('obstructive sleep apnea*' OR 'sleep apnea syndrome*' OR 'sleep hypopnea*' OR 'sleep apnea*' OR 'hypersomnia with periodic respiration' OR 'sleep disturbed breathing' OR 'sleep disordered breathing') AND ((screening*' OR 'diagnos*') AND ('accurac*' OR 'sensitivit*')) |
|  |  |  |
|  |  |  |
|  |  |  |
|  |  |  |
