# Supplementary material for: Diagnostic value of smartphone in obstructive sleep apnea syndrome: A systematic review and meta-analysis
Source: PLoS One. 2022 May 19;17(5):e0268585. doi: 10.1371/journal.pone.0268585 (PMC9119483; doi:10.1371/journal.pone.0268585)
Supplement: S2 Table — (DOCX) [file pone.0268585.s003.docx]

**S2 Table. Study characteristics.**

| **Study** | **Design** | **Number** | **Age, median (range) or mean(SD), y** | **Sex (Male/Female)** | **Apnea/hypopnea index, median (range) or mean(SD)** | **Mechanism** | **TP** | **FN** | **FP** | **TN** |
| --- | --- | --- | --- | --- | --- | --- | --- | --- | --- | --- |
| Abeyratne 2013 | Retro | 24 | NA | NA |  | Sound | 11 | 3 | 2 | 8 |
| Nakano 2014 | Pros | 50 | 47.9 (13.7) | 48/2 | 27.3 (26.1) | Sound | 25 | 4 | 4 | 17 |
| Nandakumar 2015 | Pros | 37 | 23-93 (50) | 20/17 |  | Motion | 15 | 0 | 0 | 22 |
| Bonnesen 2018 | Pros | 23 | 57.4±14.3 | 17/5 | 26.2 (21.4) | Motion and sound | 9 | 1 | 4 | 9 |
| Akhter 2018 | Pros | 85 | 50.6±16.0 | 52/33 | 42.0±28.0 | Sound | 52 | 7 | 3 | 23 |
| Swarnkar 2018 | Retro | 73 | NA | 42/31 | NA | Sound | 34 | 5 | 4 | 30 |
| Lyon 2019* | Retro | 94 | 46.5 (15.8) | 47/47 | 2.2 (15.4) | Motion | 16 | 1 | 4 | 73 |
| Lyon 2019* | Retro | 68 | 52.5 (14.0) | 46/22 | 13.9 (20.5) | Motion | 32 | 2 | 1 | 33 |
| Narayan 2019 | Pros | 59 | 52.9±15.1 | 39/20 | 30.0±32.0 | Sound | 31 | 2 | 9 | 17 |
| Tiron 2020* | Pros | 128 | 49±15 | 66/62 | 13±18 | Motion and sound | 35 | 4 | 12 | 77 |
| Tiron 2020* | Pros | 120 | 52±14 | 82/38 | 20±20 | Motion and sound | 53 | 7 | 12 | 48 |
| Chang 2020 | Pros | 56 | NA | NA | NA | Motion and oximeter | 42 | 3 | 0 | 11 |
| Pinheiro 2020 | Pros | 304 | 55±14 | 169/137 | 35.3±30.1 | Oximeter | 139 | 12 | 17 | 136 |
| Abbreviations: Pros, Prospective; Retro, Retrospective; NA, Not available; TP, True positive; FP, False positive; FN, False negative; TN, True negative | | | | | | | | | | |
| *These papers were subjected to individual subgroup analyses. | | | | | | | | | | |
