# Supplementary material for: Diagnostic value of smartphone in obstructive sleep apnea syndrome: A systematic review and meta-analysis
Source: PLoS One. 2022 May 19;17(5):e0268585. doi: 10.1371/journal.pone.0268585 (PMC9119483; doi:10.1371/journal.pone.0268585)
Supplement: S3 Table — (DOCX) [file pone.0268585.s004.docx]

**S3 Table. Methodological qualities of all included studies.**

| Reference | Risk of Bias | | | | Concerns about Application | | |
| --- | --- | --- | --- | --- | --- | --- | --- |
|  | Patient Selection | Index test | Reference standard | Flow and timing | Patient selection | Index test | Reference Standard |
| Abeyratne 2013 | Low | Low | Low | Low | Low | Low | Low |
| Nakano 2014 | Low | Low | Low | Low | Low | Low | Low |
| Nandakumar 2015 | Low | Low | Unclear | Low | Low | Low | Low |
| Bonnesen 2018 | Low | Low | Unclear | High | Low | Low | Low |
| Akhter 2018 | Low | Low | Low | Low | Low | Low | Low |
| Swarnkar 2018 | Unclear | Low | Low | Low | Low | Low | Low |
| Lyon 2019 | Low | Low | Unclear | Low | Low | Low | Low |
| Narayan 2019 | Low | Low | Unclear | Low | Low | Low | Low |
| Tiron 2020 | Unclear | Low | Low | Low | Low | Low | Low |
| Chang 2020 | Low | Low | Low | Low | Low | Low | Low |
| Pinheiro 2020 | Low | Low | Unclear | Low | Low | Low | Low |
