# Supplementary figures and images for: Diagnostic value of smartphone in obstructive sleep apnea syndrome: A systematic review and meta-analysis
Source: PLoS One. 2022 May 19;17(5):e0268585. doi: 10.1371/journal.pone.0268585 (PMC9119483; doi:10.1371/journal.pone.0268585)

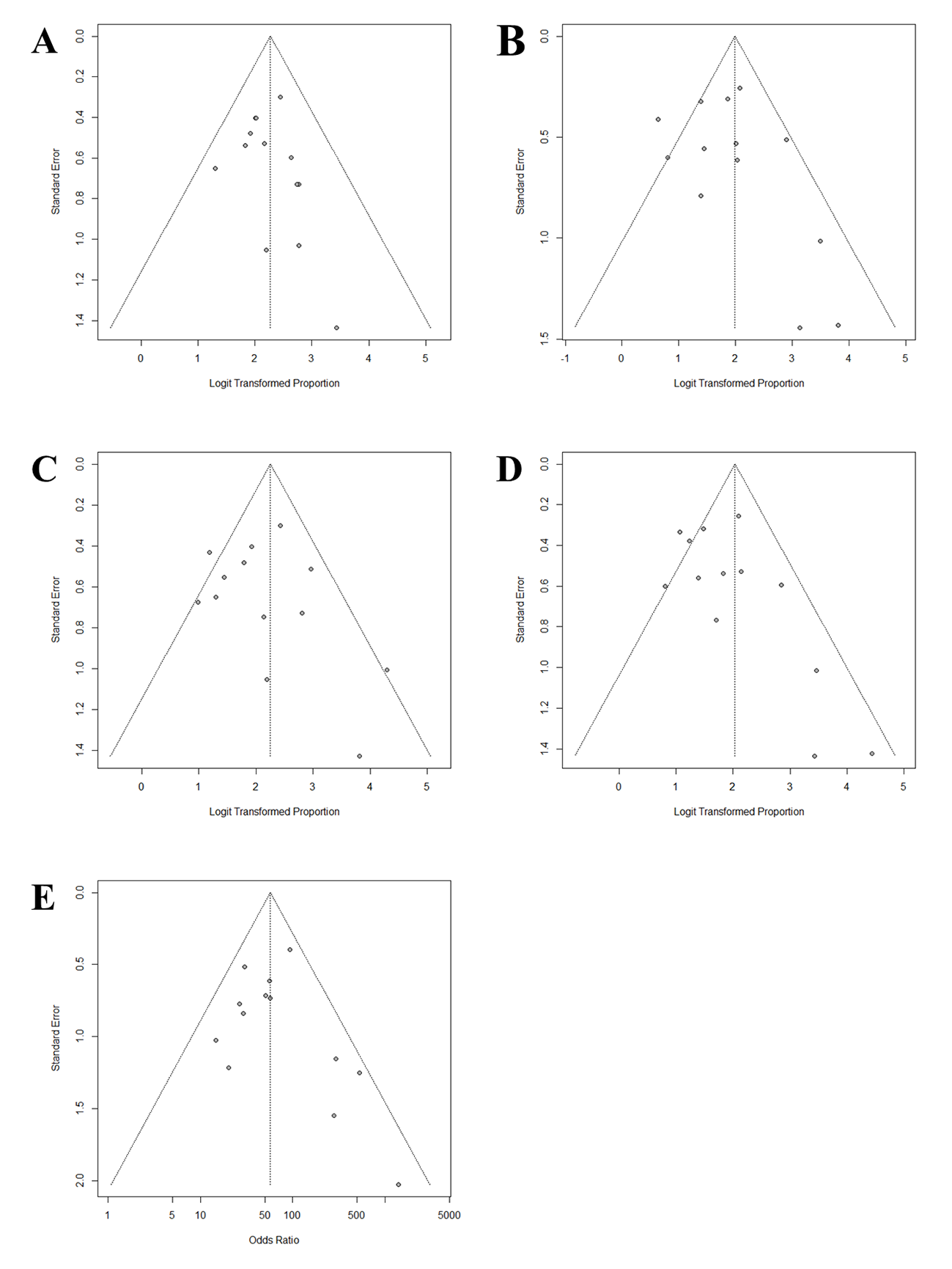

Supplement: S1 Fig — Begg’s funnel plot analyses for sensitivity (A), specificity (B), negative predictive value (C), positive predictive value (D), and diagnostic odds ratio (E). (TIF) [file pone.0268585.s005.tif]

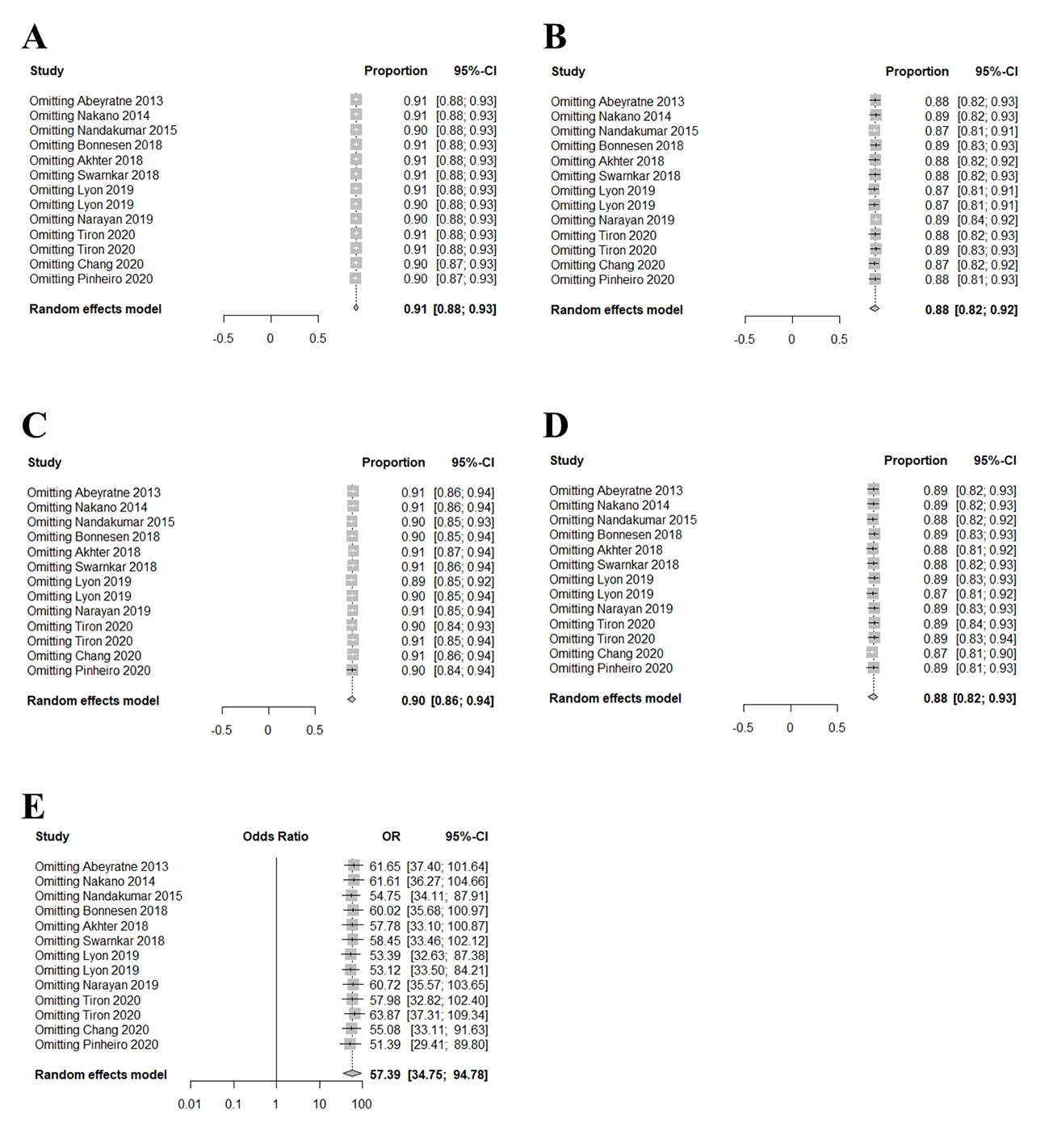

Supplement: S2 Fig — Sensitivity analyses for sensitivity (A), specificity (B), negative predictive value (C), positive predictive value (D), and diagnostic odds ratio (E). (TIF) [file pone.0268585.s006.tif]
